# Cognitive dysfunction in amyotrophic lateral sclerosis: can we predict it?

**DOI:** 10.1007/s10072-021-05188-0

**Published:** 2021-03-27

**Authors:** Fabiola De Marchi, Claudia Carrarini, Antonio De Martino, Luca Diamanti, Antonio Fasano, Antonino Lupica, Mirella Russo, Simone Salemme, Edoardo Gioele Spinelli, Alessandro Bombaci, on behalf of SIgN

**Affiliations:** 1 ALS Center & Department of Neurology, Maggiore della Carità Hospital, University of Piemonte Orientale, Novara, Italy; 2 Department of Neuroscience, Imaging and Clinical Sciences, G. D’Annunzio University, Chieti, Italy; 3 Institute of Neurology, University “Magna Graecia”, Catanzaro, Italy; 4 IRCCS Mondino Foundation, Pavia, Italy; 5 Department of Neurology, “V.Fazzi” Hospital, A.S.L. Lecce, Lecce, Italy; 6 Department of Biomedicine, Neuroscience and Advanced Diagnostic, University of Palermo, Palermo, Sicily Italy; 7 Interdepartmental Center of Neuroscience and Neurotechnologies (CfNN), University of Modena and Reggio Emilia, Modena, Italy; 8 Department of Neurology and Neuroimaging Research Unit, Division of Neuroscience, IRCCS Ospedale San Raffaele and Vita-Salute San Raffaele University, Milan, Italy; 9 “Rita Levi Montalcini” Department of Neuroscience, University of Turin, Turin, Italy

**Keywords:** ALS, Cognitive impairment, Cognitive alteration, Cognitive dysfunction, FTD, ALS-ci, ALS-bi, ALS-cbi, Biomarker, Pre-clinical

## Abstract

**Background and aim:**

Amyotrophic lateral sclerosis (ALS) is a progressive neurodegenerative disorder characterized by the degeneration of both upper and lower motoneurons in the brain and spinal cord leading to motor and extra-motor symptoms. Although traditionally considered a pure motor disease, recent evidences suggest that ALS is a multisystem disorder. Neuropsychological alterations, in fact, are observed in more than 50% of patients: while executive dysfunctions have been firstly identified, alterations in verbal fluency, behavior, and pragmatic and social cognition have also been described. Detecting and monitoring ALS cognitive and behavioral impairment even at early disease stages is likely to have staging and prognostic implications, and it may impact the enrollment in future clinical trials. During the last 10 years, humoral, radiological, neurophysiological, and genetic biomarkers have been reported in ALS, and some of them seem to potentially correlate to cognitive and behavioral impairment of patients. In this review, we sought to give an up-to-date state of the art of neuropsychological alterations in ALS: we will describe tests used to detect cognitive and behavioral impairment, and we will focus on promising non-invasive biomarkers to detect pre-clinical cognitive decline.

**Conclusions:**

To date, the research on humoral, radiological, neurophysiological, and genetic correlates of neuropsychological alterations is at the early stage, and no conclusive longitudinal data have been published. Further and longitudinal studies on easily accessible and quantifiable biomarkers are needed to clarify the time course and the evolution of cognitive and behavioral impairments of ALS patients.

## Introduction

Amyotrophic lateral sclerosis (ALS) is the most common motor neuron disease (MND) and is characterized by the progressive loss of upper and lower motor neurons causing weakness of bulbar, limb, trunk, and respiratory muscles [1]. Around 10% of cases of ALS is familial (FALS), while 90% is sporadic (SALS) [2]. In 2 out of 3 ALS patients, we observe a spinal outbreak and in 1 out of 3 a bulbar onset of disease, and only in 1% of cases, there is a respiratory onset [3]. Pathophysiology is unknown, phenotypes are different, and there are no effective treatments [1].

Diagnosis is mainly based on clinical and neurophysiological grounds (applying El Escorial criteria [4]), though some encouraging results are obtained from biomarkers studies [5].

Although for a long time ALS was considered a disease confined to motor neurons, nowadays it is established to be a multisystemic syndrome [6], and cognitive domains are involved in at least half of patients [1]. In 2017, Strong and colleagues described new criteria to classify cognitive and behavioral alterations in ALS patients, using the term frontotemporal spectrum disorder (ALS-FTSD) [7]. They identified a continuum from ALS cognitively normal (ALS-cn) to ALS with frontotemporal dementia (ALS-FTD) that includes ALS with behavioral impairment (ALSbi), ALS with cognitive impairment (ALSci), and ALS with combined cognitive and behavioral impairment (ALS-cbi). The identification of biomarkers able to detect a pre-clinical cognitive involvement would be important to better classify patients both for clinical management and for clinical trial recruitment and follow-up.

Therefore, the current review focuses on the up-to-date state of art of neuropsychological tests used to detect cognitive impairment in ALS patients and on promising non-invasive biomarkers of pre-clinical cognitive impairment.

## Neuropsychological profile in ALS

**Fig. 1:**
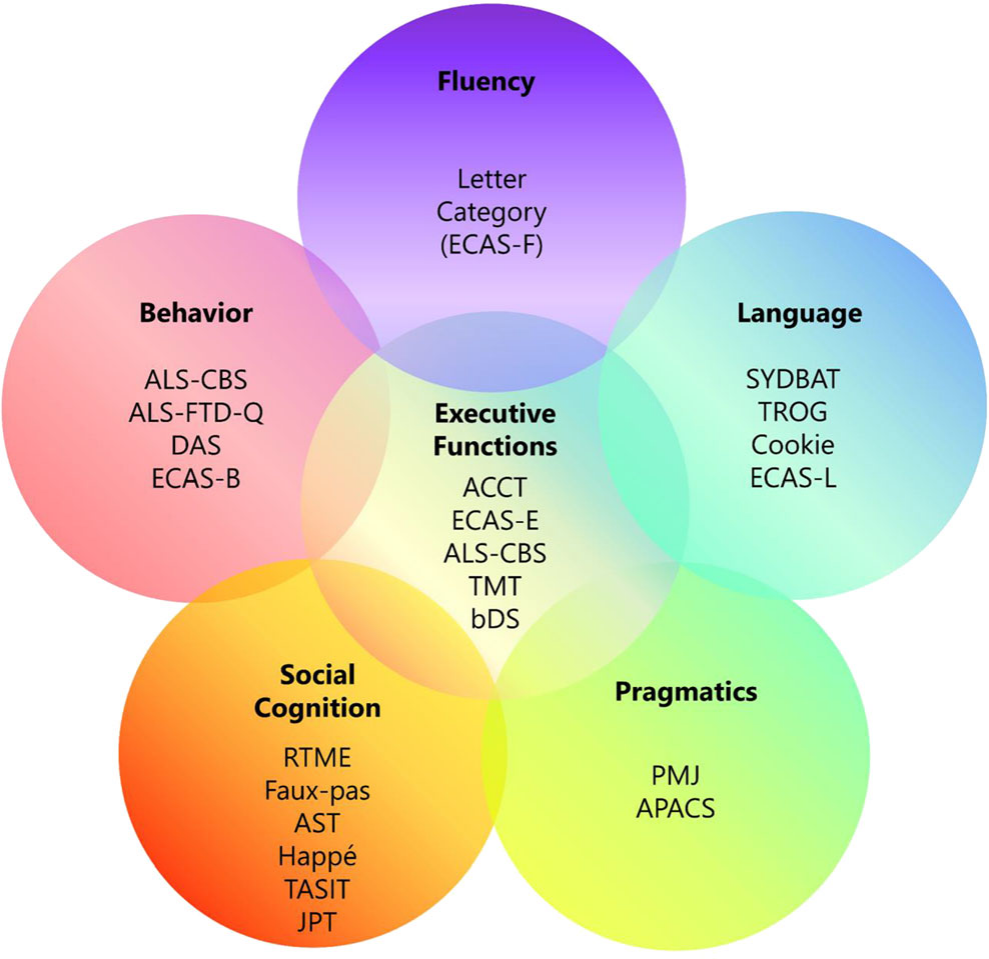
The main affected domains in patients with ALSci, ALSbi, and ALS-FTD and some available diagnostic tools. The disposition of the cognitive domains reflects the partial overlap of the underlying cognitive processes. The central position of the executive functions is due to the key role, for dysexecutive symptoms, in affecting every other cognitive domain, as well as the behavior. According to Strong criteria, to detect the presence of an ALSci clinical picture, the assessment of three “main” domains is crucial: verbal fluency, executive functions, and language skills. However, social cognition and pragmatics deficits are proxy features of executive and language impairment, respectively

Up to 40% of ALS patients exhibit mild to moderate neuropsychological alterations, which tend to be mainly observed in the bulbar phenotype, while nearly 15% of ALS cases fulfill the criteria for the behavioral variant of frontotemporal dementia (bv-FTD) [8]. The main cognitive impairment observed in ALS is represented by executive dysfunctions (Fig. 1), with verbal fluency deficit, expression of dorsolateral prefrontal alterations [9]. Executive disorders, which are a well-established measure of disease management and progression [10], include processes of inhibition, cognitive flexibility, prolonged attention, and working memory. A recent study emphasized as two specific executive sub-functions (e.g., set-shifting and initiation) seems to show greater impairment in non-demented ALS patients [11]. Another cognitive domain frequently impaired is language, although it may be difficult to disentangle fluency and language deficits [12]. Conversely, memory impairment is slightly less described in ALS, and it tends to appear in an advanced stage of disease, with hippocampal structural alterations [13].

Furthermore, social cognition (SC) and theory of mind (ToM), characterized by severe difficulties in the recognition and processing of emotions, are other features frequently affected in ALS [14]. Apathy is the most common neuropsychiatric symptom, also related to a negative prognosis, which occurs in about 50% of ALS cases. Executive, emotional, and initiation apathy [15] represent three forms of the same disturbance: executive apathy relates to a loss of attention, planning, and organization; emotional apathy, with emotional neutrality or indifference; and initiation apathy concerns a lack of motivation for the production of thoughts. To date, initiation apathy is considered the typical subtype commonly reported in ALS. The early presence of apathy has been confirmed also by recent studies [16, 17] and was reported to be directly correlated with depression severity [16], poor quality of life [16], and with widespread microstructural changes within the motor and non-motor white matter fibers, with main involvement of the corpus callosum and in the left thalamus [17]. Finally, the presence of behavioral disturbances includes also disinhibition, depression, stereotyped behaviors, and dietary changes [7]. Furthermore, the appearance of depression, although it can be justified by the patient negative prognosis, seems to be evident at least 1 year before the disease onset, suggesting a role of mood disorder in ALS pathogenesis [18]. Therefore, in order to firstly detect subtle cognitive and behavioral changes, recent efforts are being made to find suitable neuropsychological batteries for this purpose.

### Neuropsychological assessment

Great efforts have been directed towards a refined detection of cognitive deficits, in past few years. Specifically, since the new criteria from Strong et al. [7] emphasize the relevance of prodromal changes in cognition and behavior, many studies have focused on the research of new, accurate neuropsychological tools, which ought to be suitable also for speech and motor impaired patients.

Being the presence of executive deficits well established, also in ALS patients without a clear picture of cognitive impairment [7], few recent works [19] directly addressed its detection. Indeed, a new neuropsychological assessment, the Arrows and Colors Cognitive Test (ACCT), which is eye-tracking based in order to bypass verbal and motor disabilities, showed its utility in evaluating cognitive flexibility and also in discriminating between ALS patients and healthy controls (HC) [19]. Moreover, well-established screening batteries like Edinburgh Cognitive and Behavioral ALS Screen (ECAS) [20] and ALS Cognitive Behavioral Screen (ALS-CBS) [21], as well as more specific tests Trail Making Test and backward Digit Span, have also proven their efficacy and accuracy. The presence of verbal fluency deficits can be assessed in clinical practice by means of letter and category fluency tests, or by the specific ECAS item and corrected by the Verbal Fluency Index [20]. As enlightened by a recent study [22] on a cohort of non-demented patients, including ALS, ALS-bi, and ALS-ci cases, the presence of verbal fluency deficits appears to be highly predictive for TDP-43 deposition in specific brain areas (Broadmann area 9, 41, 44).

Several evidences are being gathered on different aspects of language impairment in ALS. Word retrieval deficits have been commonly outlined using both verbal fluency and confrontation naming tests. However, verbal fluency is a well-known marker of executive dysfunction too, indicating as both executive and language processes may be involved in the final performance on this task [12].

Currently, given the assumption that FTD may present with a clinical picture of primary progressive afasia (PPA), typically non-fluent/agrammatic (nfvPPA), or semantic variants (svPPA), the presence of a perfect overlap with ALS-related pictures of nfvPPA and svPPA is not clear-cut. This issue has been addressed by Long et al. [23], who compared language performances of ALS, nfvPPA, svPPA, and HC, using Sydney Language Battery (SYDBAT) [24] and Test for Reception of Grammar (TROG) [25]. The presence of a degree of language impairment was very high in the FTD-ALS (88.5%). However, two distinct patterns were observed: a mild mixed phenotype, with both semantic and syntactic deficits, and a nfvPPA-like pattern, with more pronounced syntactic impairment [23]. Post hoc analysis also showed a difference between scores at SYDBAT and TROG between ALS-cn and HC [23], confirming the presence of subtle, not clinically relevant, language deficits also in patients with ALS who do not meet criteria for ALS-ci or ALS-FTD.

One more expanding field of research in ALS patients is represented by SC abnormalities, which are multi-faceted and complex, encompassing both cognitive and affective aspects [26]. As highlighted in a recent review [26], SC deficits in ALS range from ToM abnormalities, to lack of empathy and changes in social behavior. With respect to diagnostic tools, Reading the Mind in the Eyes [27] and the Faux-pas Test are indeed the most well-known tests assessing ToM [7], and the Animated Shapes task [28], which provides moving stimuli, has also been used in a recent study [29]. Additionally, performance on understanding thoughts and feelings of cartoon characters (Happé cartoons) is also compromised in ALS. By contrast, other studies revealed opposing results for the preserve abilities in making social inferences using dynamic videos, The Awareness of Social Inference Test (TASIT) or in judging others’ preferences (Judgment of Preference task) [30].

On the other hand, self-reported and partner-reported interviews can provide information on empathy and social behavior changes. Intriguingly, a recent study by Bambini et al. [29] addressed another underestimated aspect of ALS, which straddles between SC and pragmatic fields: the impairment of verbal humor comprehension at the Phonological and Mental Jokes [31]. Furthermore, a prominent role for pragmatic impairment was observed, while scores at tests assessing executive and ToM abilities did not show a significant predictive role, in opposition to previous studies. Therefore, evaluation of pragmatic abilities could be considered in neuropsychological assessment of ALS patients, using the Assessment of Pragmatic Abilities and Cognitive Substrate (APACS) test [32] or other diagnostic tools.

As regards behavioral changes, a recent multicenter study [33] examined Japanese ALS patients with and without FTD in order to characterize cognitive and behavioral dysfunctions. The ALS-FTD-Questionnaire (ALS-FTD-Q) was assessed for behavioral screening, whereas the Montreal Cognitive Assessment (MoCA) and the Frontal Assessment Battery (FAB) were administered to evaluate cognitive performances, finding as behavioral changes were less frequent compared to cognitive deficits in ALS patients.

Moreover, the presence of apathy may be tested using the Dimensional Apathy Scale (DAS), a validated and independent of physical disability battery, combining the three previously reported subtypes of apathy. A recent research [34] showed a strict association between initiation and emotional apathy with executive and emotional cognitive dysfunctions, respectively, suggesting the presence of possible common underlying mechanisms among those neuropsychological tasks. Finally, an impact of depression on global performance at ECAS, as well as on executive functions [35], has been reported in ALS patients.

### Cognitive profile in C9Orf72 carriers

Carriers of C9Orf72 hexanucleotide expansion (C9+) are known to present with a “subphenotype” of ALS. Specifically, an earlier onset of the disease, a greater family history for ALS and/or FTD, and a greater degree of cognitive and behavioral impairment have been reported [36, 37]. Converging evidences pointed towards the existence, in pre-symptomatic carriers (C9+pc), of slight cognitive changes [38–44]. The earliest alterations (i.e., denomination and executive deficits) have been observed up to 5 years before the expected onset of motor deficits, as reported by a large multicenter study [40]. On the other hand, contrasting results emerged, over the years, on the specific cognitive domains and functions affected at the pre-symptomatic stage. Specifically, Ryan et al. found poor performances, in C9+pc, at cognitive inhibition tasks (e.g., the Hayling Sentence Completion Test, HSCT) [38]. Two studies reported a verbal memory impairment in C9+pc compared to healthy controls [41, 43]. Praxis deficits were also observed [43], as well as impaired letter fluency [44], along with deficits at the Stroop test [42].

As regards symptomatic C9+, a more rapid cognitive decline has been observed compared to non-carriers [45]. Prominent executive alterations, shown by verbal fluency, Stroop test, and Brixton spatial anticipation tasks, seem to characterize this phenotype. Difficulties in timed tasks, as well as in complex attentive tests, are also frequently found [46]. However, a relative sparing of memory (except for working memory) and visuo-spatial domains is commonly reported [47]. Even though language is progressively affected during the disease, and PNFA represents the most common phenotype among PPA variants, speech impairment is not a prevalent feature at C9+ symptomatic stage [48, 49].

In behavioral phenotype of C9+, the presenting symptoms commonly include disinhibition, apathy, and anxiety [50]. Psychotic disturbances, especially paranoid delusions and hallucinations, may represent a major hallmark of C9+ patients [48, 51].

Hence, despite a clear agreement on the existence of typical cognitive and behavioral abnormalities in C9+ individuals, further investigations are needed to better define their features, especially for the pre-symptomatic stage, also in accordance with the most recent diagnostic criteria [7].

### Progression of cognitive dysfunction

Nowadays the undefined issue concerns whether the cognitive impairment is stable or progressive over the ALS trajectory. In the literature, few heterogeneous studies are proposed in order to investigate this theme: while some authors used a cross-sectional approach demonstrating a correlation between clinical stage and cognitive disturbances [52, 53], other authors observed the stability [54, 55] or the evolution of cognitive dysfunction during a longitudinal follow-up [56, 57].

Particularly, the cognitive status worsens at the same level as the motor disability measured by ALS Functional Rating Scale–Revised scale [56, 57]. The longitudinal design is the most rigorous methodological approach in order to understand the trajectory of cognitive impairment, even though the difficulties are related to the low sample size [54] and the short duration of follow-up [56] considering the rarity and the poor prognosis of ALS patients. An additional issue regards the choice of specific but manageable tools to longitudinally test patients. Kasper et al. evidenced the initial presence of cognitive impairment that subsequently disappeared at the following time points, likely due to the starting difficulty to understand the neuropsychological battery [55]. At the moment, ECAS is the gold standard even if a practice effect can be noticed [58]. When the cohort was consistent and the follow-up adequate, it seems that cognitive profile changed over time with a direct influence on motor progression and final prognosis. Particularly patients characterized by normal status at baseline can develop cognitive dysfunctions during the follow-up [57].

## Role of genetic mutations

The majority of ALS and FTD cases are sporadic, with no positive family history for neurodegenerative and psychiatric diseases, although around 15–20% of ALS cases are inherited, usually with an autosomal dominant transmission [59]. To date, based on the definition of the Online Mendelian Inheritance in Man [60], 5 ALS-FTD genes—namely, C9Orf72 [61], CHCHD10 [62], SQSTM1 [63], TBK1 [64], and CCNF [65]—have been recognized along with roughly 30 genes associated to ALS-FTD.

These represented the major risk factors for developing familial forms of ALS-FTD; all these genes have an autosomal dominant pathway of transmission and an adult onset. Also, there are other genes which have been characterized as causative for ALS and also described in pure FTD or in cases of ALS and cognitive involvement: they are TARDBP [66], with adult onset and AD pattern, FUS, characterized by a possible juvenile onset with AD or AR pattern [67] and OPTN [68].

With the discovery of several mutations associated to cognitive impairment in ALS, several studies have begun to characterize the different phenotypes associated with these genes in terms of epidemiology, clinical presentation, imaging, and pathology. In particular, the current idea of considering ALS and FTD as part of the same disease spectrum is sustained by converging mechanisms of neurodegeneration involving RNA processing, oxidative stress, protein aggregation, and autophagy, showing as common mechanisms are responsible for susceptibilities specific to neuronal classes [69, 70].

In our opinion, the published works on genetic involvement in ALS demonstrated that some mutations, first of all the C9Orf72 one, can be considered biomarkers able to predict the development of cognitive impairment in ALS patients.

Consequently, in clinical practice, the application of next generation sequencing—as whole exome or whole genome sequencing—looking for genetic mutation in cognitive altered ALS patients has a potentially strong impact. It is important to consider, however, that not the totality of mutation carriers develop cognitive impairment with consequent clinical and ethical implications.

## Fluid biomarkers

During the last two decades, lots of biomarkers detectable in fluids and tissues have been evaluated in MNDs. Some of them are useful in clinical trial recruitment, and others have a diagnostic or a prognostic or a predictive role. The most recently studied markers are neurofilaments, both light (NfL) and heavy chains (pNfH); they are now becoming a widely accepted prognostic biomarker for ALS and other neurodegenerative diseases [71]. Unfortunately, despite the great attention put on biomarkers, only few studies showed the simultaneous evaluation of cognitive profile and humoral biomarkers in ALS, and no published studies analyzed tissue biomarkers and cognitive profile together. Most of those studies used ECAS in order to evaluate the cognitive profile. Thompson and colleagues [72] measured the levels of chitinase proteins Chitotriosidase-1 (CHIT1), Chitinase-3-like protein 1 (CHI3L1), and Chitinase-3-like protein 2 (CHI3L2) in cerebrospinal fluid (CSF) in ALS, founding only a weak association between higher CHI3L1 levels and worst ECAS score. Gala et al. reported decreased levels of soluble β fragment of amyloid precursor protein (sAPPβ) and increased levels of CHI3L1 in CSF in the ALS-FTD spectrum compared to controls [73]. Moreover, the ratio sAPPβ/ CHI3L1 correlated with cortical atrophy in frontotemporal regions in ALS and FTD. Unfortunately, none of these biomarkers correlated with the degree of cognitive and/or behavioral profile. Ahmed et al. [74] showed that hormones involved in food assumption and storage could have not only a prognostic role in ALS but can be also markers of cognitive impairment. In particular, the more the cognitive profile is altered, using the Addenbrooke’s Cognitive Examination-Revised (ACE-R), the lower levels of neuropeptide Y are and the higher levels of leptin serum are. In a recent Australian work [75], authors did flight mass spectrometry on plasma of 24 ALS patients, and they assessed cognitive profile using the ACE-III. They observed that the levels of 20 proteins were different between ALS patients with and without cognitive impairment. Contrarily, some authors showed no correlation between CSF and plasma levels of NfL and pNfH and cognitive alterations [72, 73, 76]; others observed no correlation between cognitive impairment and, respectively, brain-derived neurotrophic factor (BDNF) serum levels [77] and sβAPP [73].

Limitations of these studies include restricted number of studies concerning the simultaneous evaluation of biological biomarkers and cognitive profile, heterogeneity in scales used to evaluate cognitive impairment, scarce use of Strong criteria [7] of classification, cross-sectional analyses or short period of follow-up, lack of evaluation of fluids and tissue biomarkers before the development of cognitive impairment, and measure of biomarkers in CSF rather that in blood.

These works demonstrated that there is a lack in the evaluation of biomarkers predictive of cognitive impairment, useful to predict progression of disease, to set up a right follow-up and to screen patients to recruit in clinical trials.

## Neuroimaging as a biomarker of cognitive/behavioral impairment

In recent years, several studies have aimed to elucidate the neuroimaging signatures of cognitive and behavioral impairment in the ALS-FTD spectrum. Magnetic resonance imaging (MRI) was the most commonly applied tool in these studies, particularly using advanced techniques able to study even subtle brain structural and functional alterations, although also positron emission tomography (PET) techniques have been increasingly explored in such context [78]. However, most of these studies assessed neuroimaging correlates of impairment in specific cognitive/behavioral scales, rather than providing a systematic characterization of ALS patients with mild cognitive and/or behavioral impairment according to the recently revised Strong criteria [7] or other previous classification systems [79, 80]. Moreover, there is a dramatic lack of longitudinal studies, which are necessary to support a role of these tools for predicting the future development of cognitive decline in ALS.

### Magnetic resonance imaging

On visual inspection of conventional MR images, global brain atrophy is relatively mild or even undetectable in patients with ALS [81]. Therefore, the assessment of regional differences in gray matter (GM) loss by voxel-based or surface-based morphometry analyses is likely more important to characterize the underpinnings of cognitive alterations in ALS patients. Although, as expected, ALS-FTD patients have shown the most severe atrophy involving widespread frontotemporal cortical areas and subcortical regions (e.g., the caudate nucleus) [82, 83], significant GM loss in extensive frontal, temporal, and—to a lesser degree—more posterior cortical brain regions has been observed also in patients with subtle cognitive and/or behavioral impairment [82, 84]. Among studies which assessed specifically ALS-cbi patients compared with ALS-cn, greater thinning of inferior frontal, temporal, cingulate, and insular cortices was associated with worse performance in executive, language, verbal fluency, social cognition, and episodic memory task [84, 85], in contrast with the focal cortical thinning observed in the dorsal motor cortex of ALS-cn patients [86]. Reduced volumes of the precuneus [87] and subcortical structures, including the thalamus and the amygdala [88], have also been shown in ALS-ci compared with ALS-cn. In particular, basal ganglia alterations have been associated with greater behavioral impairment in ALS patients [89].

Diffusion tensor imaging (DTI) studies have also demonstrated distinctive microstructural abnormalities of the white matter (WM) in ALS with mild cognitive/behavioral impairment, in terms of diffusivity alterations within extra-motor (i.e., associative) tracts, including the uncinate, cingulum, superior longitudinal fasciculus, and fornix [83, 86], correlating with the severity of cognitive and behavioral alterations [17]. These findings, as well as a recent network-based study correlating the structural disruption of frontal networks with executive dysfunction in ALS patients [90], support the notion of cognitive impairment in this condition as the result of a “disconnection syndrome” occurring when extra-motor brain WM tracts are pathologically involved. Although longitudinal progression of such extra-motor WM alterations has been shown by several studies [91, 92], and even a sequential cognitive staging system based on cross-sectional DTI findings has been proposed for ALS patients [13], further studies are needed to test the theory that neuroanatomical progression of pathology to widespread frontotemporal regions might parallel worsening cognitive functions.

Functional MRI holds the promise to further elucidate the underpinnings of ALS-cbi, as executive dysfunction and behavioral disturbances in ALS have been associated with disrupted functional connectivity in frontoparietal, salience, and executive networks [93, 94]. However, to date, only one study has specifically assessed functional rearrangements in patients fulfilling ALS-cbi criteria, showing more severe rearrangements in inferior parietal and cerebellar networks, compared with ALS-cn [95].

### PET studies

According to the 2018 European Association of Nuclear Medicine recommendations for the use of 18F-FDG PET in neurodegenerative cognitive impairment and dementia [96], 18F-FDG PET may have diagnostic and prognostic value in selected cases; however, its utility for general clinical purposes in ALS-cbi patients remains controversial. Therefore, panelists suggest 18F-FDG PET to only be used for research purposes, not recommending it for clinical use [96].

While no definite 18F-FDG PET biomarker for future cognitive decline has been identified yet, data from few cross-sectional studies show how changes in cerebral glucose metabolism also occur in extra-motor regions. Canosa et al. [97] showed a decreasing gradient of frontal lobe metabolism in ALS patients, going from ALS-cn to ALS-FTD cases. Their results suggest a continuum of hypometabolism paralleling increasing cognitive decline. Furthermore, a study [87] found changes in cerebral glucose metabolism even in ALS patients without cognitive and behavioral deficits, compared to healthy individuals. Interestingly, the authors showed how hypermetabolism in the right and left hippocampus as well as in the left parahippocampal gyrus was associated with poorer scores in episodic memory tests, and hypermetabolism in the left fusiform gyrus was associated with impaired ToM [98]. The authors suggested a deleterious neuronal and/or astrocytic process to be the cause of the hypermetabolism.

Impaired GABAergic neurotransmission shown by reduced uptake of 11C-flumazenil has also been correlated with poor performance at executive tasks in ALS patients [99]. The recent development of PET ligands which target glutamatergic synapses might provide further insights regarding excitotoxic alterations in ALS and their relationship with cognitive/behavioral symptoms.

### Imaging in C9Orf72 carriers

MRI has been also applied to the study of genetic forms of ALS, such as C9Orf72 mutations, the best-known molecular link between ALS and FTD. Compared to non-carriers, ALS patients with a C9Orf72 pathological expansion exhibit greater cortical and subcortical brain atrophy (notably, involving parieto-occipital cortical regions, thalami, and cerebellar structures), more diffuse involvement of white matter pathways, and distinctive alterations of visual and thalamic functional networks [100–102]. Subtle structural and functional imaging alterations in a similar pattern have also been reported in young adult C9+pc, compared with familial non-carriers of similar age, suggesting early rearrangements occurring long before the development of motor and/or cognitive symptoms [41, 103].

In PET studies, an interesting work conducted on C9Orf72 ALS-FTD patients showed how hypometabolism in the thalami may discriminate C9Orf72 mutation carriers from non-carriers [104]. Bilateral thalamic relative hypometabolism has also been observed in C9+pc compared with C9- healthy controls, as well as hypometabolism in the frontotemporal and insular cortex and the basal ganglia [105].

## Neurophysiological approaches

Recent anatomopathological studies in ALS subjects suggested that misfolded TDP-43 progressively spreads along corticofugal axonal pathways from the motor cortex to other cortical and subcortical regions [106]. These results pave the way to the novel concept of disconnection syndromes [107]; it has been suggested, in fact, the existence of shared patterns of connectivity impairment across neurological disorders [107]. A recent position paper hypothesized that biomarkers indicative of networks impairment may be altered before the beginning of axonal degeneration: thus, the evaluation of network dysfunction has the potential to be developed as a predictive biomarker of cognitive deterioration in ALS [108].

Advanced neurophysiological techniques have been used as a tool to investigate the functioning of specific brain networks [109]. Both the mismatch negativity (MMN) paradigm and the error detection task provided quantitative measures that correlate with cognitive change in ALS patients. When subjects with ALS underwent the mismatch-negativity protocol with a high-density EEG (HD-EEG), they showed significant increase in power of the left posterior parietal, central, and dorsolateral prefrontal cortices as compared with HC [110]; these changes strikingly correlated with clinical scores aimed to evaluate cognitive flexibility [110]. ALS patients enrolled in another study showed that MMN waveforms were attenuated in early onset with an increased average delay [111]; this enhanced delay strikingly correlated with changes in the Stroop test, a neuropsychological test designed to evaluate the functioning of the anterior cingulate cortex, and the dorsolateral prefrontal cortex [111].

ALS patients engaged in Go-NoGo trial demonstrated a significant different activation of the P3 wave in the left posterior parietal and insular cortex, and this difference negatively correlated with a clinical score indicating behavioral inhibition [112].

Focusing on brain networks, an innovative HD-EEG study pointed out that ALS patients showed a nonspecific and widespread increasing in connectivity pattern among brain areas [113]. The grouping of the complex connectivity pattern into distinct brain networks gave the opportunity to selectively study the specific involvement of frontoparietal and frontotemporal networks, demonstrating an increased co-modulation in the theta-frequency band in the frontal-parietal network in ALS [113]. Furthermore, the co-modulation of brain waves in the gamma-frequency band within the frontotemporal network has been found to be higher in ALS patients and correlated with language impairment [113].

Complementary to HD-EEG findings, transcranial magnetic stimulation (TMS) has also been used as a tool to assess the functioning of the cortex and to further investigate cortical networks [114]. In a recent study, patients suffering from ALS underwent a neurophysiological study including TMS and a complete cognitive and behavioral assessment [115]. Authors described that a lower resting motor threshold (RMT) as well as the bulbar onset were independently associated with cognitive impairment as assessed with the ACE score [115]; it suggests that cortical circuits underlying RMT generation also play a role in the development of cognitive decline. Cortex hyperexcitability as highlighted by a lower RMT, in fact, is a key feature of both ALS and FTD [116]. Same findings were replicated in a larger cohort of FTD patients using the *Mini Mental State Examination* test [117].

Looking at these data, we could conclude that these novel approaches can provide details about networks impairment in ALS patients, spanning from the columnar structure of the motor cortex to broader brain networks. Since characterization of pre-symptomatic subjects is a priority for ALS, these methods are ideal candidate biomarkers to detect cognitive impairment during the pre-clinical phase of the disease and to be used as data-driven quantitative outcomes in clinical trials. A main limitation of these findings is the lack of homogeneity of the used neuropsychological batteries as well as of the neurophysiological techniques and outcomes.

## Conclusions

During the last years, the confirmation of cognitive dysfunction in ALS patients have changed our point of view from a pure “motor” syndrome to include also “extra-motor” symptoms. Coherently, the search for candidate biomarkers of cognitive impairment has launched, including reproducible and non-invasive tests such as imaging, physiology, genetic, and biofluid measurements.

Nevertheless, the development of such biomarkers is still at the early stage of identifying measures that differ in group comparisons. This seems to be mainly due to the lack of a common assessment and evaluation of cognitive impairment, to the limited number of longitudinal studies concerning contemporary evaluation of cognitive assessment and other biomarkers, to the use of cognitive data as an associated variable rather than a primary outcome and to the very rare number of studies on pre-symptomatic patients carrying a pathogenic mutation.

In our opinion, it would be important to achieve longitudinal studies in which non-invasive biomarkers should be evaluated together with the cognitive assessment, recruiting ALS patients at the beginning of the disease. Furthermore, the cognitive assessment should be done using a batch of neuropsychological tests able to better classify patients following the currently used Strong criteria. That pursuit would be useful for patients’ deep phenotyping, subgrouping, better clinical evaluation and follow-up, and early identification of responders in future clinical trials.

## Abbreviations

ACCT: Arrows and Colors Cognitive Test
ALS-CBS: ALS Cognitive Behavioral Screen
ALS-FTD-Q: Amyotrophic Lateral Sclerosis-FrontoTemporal Dementia–Questionnaire
APACS: Assessment of Pragmatic Abilities and Cognitive Substrate
bDS: Digit Span–backward
Cookie: Cookie Theft Picture
DAS: Dimensional Apathy Scale
ECAS: Edinburgh Cognitive and Behavioral ALS Screen
ECAS-B: behavioral section of the ECAS
ECAS-E: ECAS section for the assessment of executive deficits
ECAS-F: verbal fluency section of the ECAS
ECAS-L: language section of the ECAS
Faux-pas: Faux-pas Test
Happé: Happé cartoons
JPT: Judgment of Preference task
PMJ: Phonological and Mental Jokes
RTME: Reading the Mind in the Eyes
SYDBAT: Sydney Language Battery
TASIT: The Awareness of Social Inference Test
TMT: Trail Making Test
TROG: Test for Reception of Grammar

## References

[1] Hardiman O, Al-Chalabi A, Chio A et al (2017) Amyotrophic lateral sclerosis. Nat Rev Dis Prim 3(1):17071. 10.1038/nrdp.2017.71 10.1038/nrdp.2017.71 28980624

[2] Renton AE, Chiò A, Traynor BJ (2014). State of play in amyotrophic lateral sclerosis genetics. Nat Neurosci.

[3] Chiò A, Calvo A, Moglia C, Mazzini L, Mora G (2011). Phenotypic heterogeneity of amyotrophic lateral sclerosis: a population based study. J Neurol Neurosurg Psychiatry.

[4] Ludolph A, Drory V, Hardiman O, Nakano I, Ravits J, Robberecht W, Shefner J, for The WFN Research Group On ALS/MND (2015). A revision of the El Escorial criteria-2015. Amyotroph Lateral Scler Front Degener.

[5] Lombardi V, Bombaci A, Zampedri L, Lu CH, Malik B, Zetterberg H, Heslegrave AJ, Rinaldi C, Greensmith L, Hanna MG, Malaspina A, Fratta P (2019). Plasma pNfH levels differentiate SBMA from ALS. J Neurol Neurosurg Psychiatry.

[6] Caga J, Hsieh S, Lillo P, Dudley K, Mioshi E (2019) The impact of cognitive and behavioral symptoms on ALS patients and their caregivers. Front Neurol 10. 10.3389/fneur.2019.00192 10.3389/fneur.2019.00192 PMC6421295 30915018

[7] Strong MJ, Abrahams S, Goldstein LH, Woolley S, Mclaughlin P, Snowden J, Mioshi E, Roberts-South A, Benatar M, HortobáGyi T, Rosenfeld J, Silani V, Ince PG, Turner MR (2017). Amyotrophic lateral sclerosis - frontotemporal spectrum disorder (ALS-FTSD): revised diagnostic criteria. Amyotroph Lateral Scler Front Degener.

[8] Raaphorst J, Beeldman E, De Visser M, De Haan RJ, Schmand B (2012). A systematic review of behavioural changes in motor neuron disease. Amyotroph Lateral Scler.

[9] Abrahams S (2013). Executive dysfunction in ALS is not the whole story. J Neurol Neurosurg Psychiatry.

[10] Elamin M, Phukan J, Bede P, Jordan N, Byrne S, Pender N, Hardiman O (2011). Executive dysfunction is a negative prognostic indicator in patients with ALS without dementia. Neurology..

[11] Kasper E, Schuster C, Machts J, Bittner D, Vielhaber S, Benecke R, Teipel S, Prudlo J (2015). Dysexecutive functioning in ALS patients and its clinical implications. Amyotroph Lateral Scler Front Degener.

[12] Pinto-Grau M, Hardiman O, Pender N (2018) The study of language in the amyotrophic lateral sclerosis - frontotemporal spectrum disorder: a systematic review of findings and new perspectives. Neuropsychol Rev 28(2):251–268. 10.1007/s11065-018-9375-7 10.1007/s11065-018-9375-7 29705950

[13] Lulé D, Böhm S, Müller HP, Aho-Özhan H, Keller J, Gorges M, Loose M, Weishaupt JH, Uttner I, Pinkhardt E, Kassubek J, del Tredici K, Braak H, Abrahams S, Ludolph AC (2018). Cognitive phenotypes of sequential staging in amyotrophic lateral sclerosis. Cortex..

[14] Trojsi F, Siciliano M, Russo A (2016). Theory of mind and its neuropsychological and quality of life correlates in the early stages of amyotrophic lateral sclerosis. Front Psychol.

[15] Radakovic R, Stephenson L, Colville S, Swingler R, Chandran S, Abrahams S (2016). Multidimensional apathy in ALS: validation of the dimensional apathy scale. J Neurol Neurosurg Psychiatry.

[16] Caga J, Hsieh S, Highton-Williamson E, Zoing MC, Ramsey E, Devenney E, Ahmed RM, Kiernan MC (2018). Apathy and its impact on patient outcome in amyotrophic lateral sclerosis. J Neurol.

[17] Femiano C, Trojsi F, Caiazzo G, Siciliano M, Passaniti C, Russo A, Bisecco A, Cirillo M, Monsurrò MR, Esposito F, Tedeschi G, Santangelo G (2018). Apathy is correlated with widespread diffusion tensor imaging (DTI) impairment in amyotrophic lateral sclerosis. Behav Neurol.

[18] De Marchi F, Sarnelli MF, Solara V, Bersano E, Cantello R, Mazzini L (2019) Depression and risk of cognitive dysfunctions in amyotrophic lateral sclerosis. Acta Neurol Scand 139(5). 10.1111/ane.13073 10.1111/ane.13073 30712314

[19] Poletti B, Carelli L, Faini A, Solca F, Meriggi P, Lafronza A, Ciringione L, Pedroli E, Ticozzi N, Ciammola A, Cipresso P, Riva G, Silani V (2018). The Arrows and Colors Cognitive Test (ACCT): a new verbal-motor free cognitive measure for executive functions in ALS. PLoS One.

[20] Poletti B, Solca F, Carelli L, Madotto F, Lafronza A, Faini A, Monti A, Zago S, Calini D, Tiloca C, Doretti A, Verde F, Ratti A, Ticozzi N, Abrahams S, Silani V (2016). The validation of the Italian Edinburgh Cognitive and Behavioural ALS Screen (ECAS). Amyotroph Lateral Scler Frontotemporal Degener.

[21] Woolley SC, York MK, Moore DH, Strutt AM, Murphy J, Schulz PE, Katz JS (2010). Detecting frontotemporal dysfunction in ALS: utility of the ALS Cognitive Behavioral Screen (ALS-CBS). Amyotroph Lateral Scler Off Publ World Fed Neurol Res Gr Mot Neuron Dis.

[22] Gregory JM, McDade K, Bak TH, Pal S, Chandran S, Smith C, Abrahams S (2019). Executive, language and fluency dysfunction are markers of localised TDP-43 cerebral pathology in non-demented ALS. J Neurol Neurosurg Psychiatry.

[23] Long Z, Irish M, Piguet O, Kiernan MC, Hodges JR, Burrell JR (2019). Clinical and neuroimaging investigations of language disturbance in frontotemporal dementia–motor neuron disease patients. J Neurol.

[24] Savage S, Hsieh S, Leslie F, Foxe D, Piguet O, Hodges JR (2013). Distinguishing subtypes in primary progressive aphasia: application of the Sydney language battery. Dement Geriatr Cogn Disord.

[25] Bishop DVM (1982). Comprehension of spoken, written and signed sentences in childhood language disorders. J Child Psychol Psychiatry.

[26] Christidi F, Migliaccio R, Santamaría-García H, Santangelo G, Trojsi F (2018). Social cognition dysfunctions in neurodegenerative diseases: neuroanatomical correlates and clinical implications. Behav Neurol.

[27] Baron-Cohen S, Wheelwright S, Hill J, Raste Y, Plumb I (2001). The “Reading the Mind in the Eyes” test revised version: a study with normal adults, and adults with Asperger syndrome or high-functioning autism. J Child Psychol Psychiatry.

[28] Abell F, Happé F, Frith U (2000). Do triangles play tricks? Attribution of mental states to animated shapes in normal and abnormal development. Cogn Dev.

[29] Bambini V, Bischetti L, Bonomi CG, Arcara G, Lecce S, Ceroni M (2020). Beyond the motor account of amyotrophic lateral sclerosis: verbal humour and its relationship with the cognitive and pragmatic profile. Int J Lang Commun Disord.

[30] Strikwerda-Brown C, Ramanan S, Irish M (2019). Neurocognitive mechanisms of theory of mind impairment in neurodegeneration: a transdiagnostic approach. Neuropsychiatr Dis Treat.

[31] Bischetti L, Ceccato I, Lecce S, Cavallini E, Bambini V (2019) Pragmatics and theory of mind in older adults’ humor comprehension. Curr Psychol. 10.1007/s12144-019-00295-w

[32] Arcara G, Bambini V (2016). A test for the Assessment of Pragmatic Abilities and Cognitive Substrates (APACS): normative data and psychometric properties. Front Psychol.

[33] Watanabe Y, Raaphorst J, Izumi Y (2020). Cognitive and behavioral status in Japanese ALS patients: a multicenter study. J Neurol.

[34] Radakovic R, Stephenson L, Newton J, Crockford C, Swingler R, Chandran S, Abrahams S (2017). Multidimensional apathy and executive dysfunction in amyotrophic lateral sclerosis. Cortex..

[35] Carelli L, Solca F, Faini A (2018). The complex interplay between depression/anxiety and executive functioning: insights from the ECAS in a large ALS Population. Front Psychol.

[36] Byrne S, Elamin M, Bede P, Shatunov A, Walsh C, Corr B, Heverin M, Jordan N, Kenna K, Lynch C, McLaughlin RL, Iyer PM, O'Brien C, Phukan J, Wynne B, Bokde AL, Bradley DG, Pender N, al-Chalabi A, Hardiman O (2012). Cognitive and clinical characteristics of patients with amyotrophic lateral sclerosis carrying a C9orf72 repeat expansion: a population-based cohort study. Lancet Neurol.

[37] Consonni M, Dalla Bella E, Nigri A, Pinardi C, Demichelis G, Porcu L, Gellera C, Pensato V, Cappa SF, Bruzzone MG, Lauria G, Ferraro S (2019). Cognitive syndromes and C9orf72 mutation are not related to cerebellar degeneration in amyotrophic lateral sclerosis. Front Neurosci.

[38] Ryan M, Costello E, Doherty MA (2020). Cognitive dysfunction in pre-symptomatic C9orf72 carriers (1774). Neurology..

[39] Montembeault M, Sayah S, Rinaldi D, le Toullec B, Bertrand A, Funkiewiez A, Saracino D, Camuzat A, Couratier P, Chouly M, Hannequin D, Aubier-Girard C, Pasquier F, Delbeuck X, Colliot O, Batrancourt B, Azuar C, Lévy R, Dubois B, le Ber I, Migliaccio R (2020). Cognitive inhibition impairments in presymptomatic C9orf72 carriers. J Neurol Neurosurg Psychiatry.

[40] Rohrer JD, Nicholas JM, Cash DM, van Swieten J, Dopper E, Jiskoot L, van Minkelen R, Rombouts SA, Cardoso MJ, Clegg S, Espak M, Mead S, Thomas DL, de Vita E, Masellis M, Black SE, Freedman M, Keren R, MacIntosh BJ, Rogaeva E, Tang-Wai D, Tartaglia MC, Laforce R, Tagliavini F, Tiraboschi P, Redaelli V, Prioni S, Grisoli M, Borroni B, Padovani A, Galimberti D, Scarpini E, Arighi A, Fumagalli G, Rowe JB, Coyle-Gilchrist I, Graff C, Fallström M, Jelic V, Ståhlbom AK, Andersson C, Thonberg H, Lilius L, Frisoni GB, Binetti G, Pievani M, Bocchetta M, Benussi L, Ghidoni R, Finger E, Sorbi S, Nacmias B, Lombardi G, Polito C, Warren JD, Ourselin S, Fox NC, Rossor MN (2015). Presymptomatic cognitive and neuroanatomical changes in genetic frontotemporal dementia in the Genetic Frontotemporal dementia Initiative (GENFI) study: a cross-sectional analysis. Lancet Neurol.

[41] Lee SE, Sias AC, Mandelli ML, Brown JA, Brown AB, Khazenzon AM, Vidovszky AA, Zanto TP, Karydas AM, Pribadi M, Dokuru D, Coppola G, Geschwind DH, Rademakers R, Gorno-Tempini ML, Rosen HJ, Miller BL, Seeley WW (2017). Network degeneration and dysfunction in presymptomatic C9Orf72 expansion carriers. NeuroImage Clin.

[42] Papma JM, Jiskoot LC, Panman JL, Dopper EG, den Heijer T, Donker Kaat L, Pijnenburg YAL, Meeter LH, van Minkelen R, Rombouts SARB, van Swieten JC (2017). Cognition and gray and white matter characteristics of presymptomatic C9orf72 repeat expansion. Neurology..

[43] Bertrand A, Wen J, Rinaldi D, Houot M, Sayah S, Camuzat A, Fournier C, Fontanella S, Routier A, Couratier P, Pasquier F, Habert MO, Hannequin D, Martinaud O, Caroppo P, Levy R, Dubois B, Brice A, Durrleman S, Colliot O, le Ber I, for the Predict to Prevent Frontotemporal Lobar Degeneration and Amyotrophic Lateral Sclerosis (PREV-DEMALS) Study Group (2018). Early cognitive, structural, and microstructural changes in presymptomatic C9orf72 carriers younger than 40 years. JAMA Neurol.

[44] Lulé DE, Müller H-P, Finsel J, Weydt P, Knehr A, Winroth I, Andersen P, Weishaupt J, Uttner I, Kassubek J, Ludolph AC (2020). Deficits in verbal fluency in presymptomatic C9orf72 mutation gene carriers—a developmental disorder. J Neurol Neurosurg Psychiatry.

[45] Irwin DJ, McMillan CT, Brettschneider J (2013). Cognitive decline and reduced survival in C9orf72 expansion frontotemporal degeneration and amyotrophic lateral sclerosis. J Neurol Neurosurg Psychiatry.

[46] Boeve BF, Boylan KB, Graff-Radford NR, DeJesus-Hernandez M, Knopman DS, Pedraza O, Vemuri P, Jones D, Lowe V, Murray ME, Dickson DW, Josephs KA, Rush BK, Machulda MM, Fields JA, Ferman TJ, Baker M, Rutherford NJ, Adamson J, Wszolek ZK, Adeli A, Savica R, Boot B, Kuntz KM, Gavrilova R, Reeves A, Whitwell J, Kantarci K, Jack CR, Parisi JE, Lucas JA, Petersen RC, Rademakers R (2012). Characterization of frontotemporal dementia and/or amyotrophic lateral sclerosis associated with the GGGGCC repeat expansion in C9Orf72. Brain..

[47] Boeve BF, Gra-Radford NR (2012). Cognitive and behavioral features of c9ftd/als. Alzheimers Res Ther.

[48] Snowden JS, Rollinson S, Thompson JC, Harris JM, Stopford CL, Richardson AMT, Jones M, Gerhard A, Davidson YS, Robinson A, Gibbons L, Hu Q, DuPlessis D, Neary D, Mann DMA, Pickering-Brown SM (2012). Distinct clinical and pathological characteristics of frontotemporal dementia associated with C9Orf72 mutations. Brain..

[49] Hsiung GYR, Dejesus-Hernandez M, Feldman HH (2012). Clinical and pathological features of familial frontotemporal dementia caused by C9Orf72 mutation on chromosome 9p. Brain..

[50] Mahoney CJ, Beck J, Rohrer JD, Lashley T, Mok K, Shakespeare T, Yeatman T, Warrington EK, Schott JM, Fox NC, Rossor MN, Hardy J, Collinge J, Revesz T, Mead S, Warren JD (2012). Frontotemporal dementia with the C9Orf72 hexanucleotide repeat expansion: clinical, neuroanatomical and neuropathological features. Brain..

[51] Patel AN, Sampson JB (2015) Cognitive profile of C9orf72 in frontotemporal dementia and amyotrophic lateral sclerosis. Curr Neurol Neurosci Rep 15(9). 10.1007/s11910-015-0582-9 10.1007/s11910-015-0582-9 26198888

[52] Trojsi F, Santangelo G, Caiazzo G, Siciliano M, Ferrantino T, Piccirillo G, Femiano C, Cristillo V, Monsurrò MR, Esposito F, Tedeschi G (2016). Neuropsychological assessment in different King’s clinical stages of amyotrophic lateral sclerosis. Amyotroph Lateral Scler Front Degener.

[53] Chiò A, Moglia C, Canosa A, Manera U, Vasta R, Brunetti M, Barberis M, Corrado L, D'Alfonso S, Bersano E, Sarnelli MF, Solara V, Zucchetti JP, Peotta L, Iazzolino B, Mazzini L, Mora G, Calvo A (2019). Cognitive impairment across ALS clinical stages in a population-based cohort. Neurology..

[54] Kilani M, Micallef J, Soubrouillard C, Rey-Lardiller D, Dematteï C, Dib M, Philippot P, Ceccaldi M, Pouget J, Blin O (2004). A longitudinal study of the evolution of cognitive function and affective state in patients with amyotrophic lateral sclerosis. Amyotroph Lateral Scler Other Mot Neuron Disord.

[55] Kasper E, Zydatiss K, Schuster C, Machts J, Bittner D, Kaufmann J, Benecke R, Vielhaber S, Teipel S, Prudlo J (2016). No change in executive performance in ALS patients: a longitudinal neuropsychological study. Neurodegener Dis.

[56] Elamin M, Bede P, Byrne S, Jordan N, Gallagher L, Wynne B, O'Brien C, Phukan J, Lynch C, Pender N, Hardiman O (2013). Cognitive changes predict functional decline in ALS: a population-based longitudinal study. Neurology..

[57] Bersano E, Sarnelli MF, Solara V et al (2020) Decline of cognitive and behavioral functions in amyotrophic lateral sclerosis: a longitudinal study. Amyotroph Lateral Scler Front Degener 1–7 10.1080/21678421.2020.1771732 32484726

[58] Poletti B, Solca F, Carelli L, Faini A, Madotto F, Lafronza A, Monti A, Zago S, Ciammola A, Ratti A, Ticozzi N, Abrahams S, Silani V (2018). Cognitive-behavioral longitudinal assessment in ALS: the Italian Edinburgh Cognitive and Behavioral ALS Screen (ECAS). Amyotroph Lateral Scler Front Degener.

[59] Brenner D, Weishaupt JH (2019). Update on amyotrophic lateral sclerosis genetics. Curr Opin Neurol.

[60] Hamosh A, Scott AF, Amberger J, Bocchini C, Valle D, McKusick VA (2002). Online Mendelian Inheritance in Man (OMIM), a knowledgebase of human genes and genetic disorders. Nucleic Acids Res.

[61] van Blitterswijk M, DeJesus-Hernandez M, Niemantsverdriet E, Murray ME, Heckman MG, Diehl NN, Brown PH, Baker MC, Finch NCA, Bauer PO, Serrano G, Beach TG, Josephs KA, Knopman DS, Petersen RC, Boeve BF, Graff-Radford NR, Boylan KB, Petrucelli L, Dickson DW, Rademakers R (2013). Association between repeat sizes and clinical and pathological characteristics in carriers of C9Orf72 repeat expansions (Xpansize-72): a cross-sectional cohort study. Lancet Neurol.

[62] Consortium PMALSS, Tazelaar GHP, van Rheenen W (2018). Chchd10 variants in amyotrophic lateral sclerosis: where is the evidence?. Ann Neurol.

[63] Rubino E, Rainero I, Chiò A (2012). SQSTM1 mutations in frontotemporal lobar degeneration and amyotrophic lateral sclerosis. Neurology..

[64] van der Zee J, Gijselinck I, Van Mossevelde S (2017). TBK1 mutation spectrum in an extended European patient cohort with frontotemporal dementia and amyotrophic lateral sclerosis. Hum Mutat.

[65] Williams KL, Topp S, Yang S (2016). CCNF mutations in amyotrophic lateral sclerosis and frontotemporal dementia. Nat Commun.

[66] Majumder V, Gregory JM, Barria MA, Green A, Pal S (2018). TDP-43 as a potential biomarker for amyotrophic lateral sclerosis: a systematic review and meta-analysis. BMC Neurol.

[67] Vance C, Rogelj B, Hortobágyi T (2009). Mutations in FUS, an RNA processing protein, cause familial amyotrophic lateral sclerosis type 6. Science (80- ).

[68] Feng S, Che C, Feng S (2019). Novel mutation in optineurin causing aggressive ALS+/− frontotemporal dementia. Ann Clin Transl Neurol.

[69] Ling S-C, Polymenidou M, Cleveland DW (2013). Converging mechanisms in ALS and FTD: disrupted RNA and protein homeostasis. Neuron..

[70] Balendra R, Isaacs AM (2018). C9orf72-mediated ALS and FTD: multiple pathways to disease. Nat Rev Neurol.

[71] Zucchi E, Bonetto V, Sorarù G et al (2020) Neurofilaments in motor neuron disorders: towards promising diagnostic and prognostic biomarkers. Mol Neurodegener 15(1). 10.1186/s13024-020-00406-3 10.1186/s13024-020-00406-3 PMC7559190 33059698

[72] Thompson AG, Gray E, Bampton A, Raciborska D, Talbot K, Turner MR (2019). CSF chitinase proteins in amyotrophic lateral sclerosis. J Neurol Neurosurg Psychiatry.

[73] Illán-Gala I, Alcolea D, Montal V, Dols-Icardo O, Muñoz L, de Luna N, Turón-Sans J, Cortés-Vicente E, Sánchez-Saudinós MB, Subirana A, Sala I, Blesa R, Clarimón J, Fortea J, Rojas-García R, Lleó A (2018). CSF sAPPβ, YKL-40, and NfL along the ALS-FTD spectrum. Neurology..

[74] Ahmed RM, Phan K, Highton-Williamson E, Strikwerda-Brown C, Caga J, Ramsey E, Zoing M, Devenney E, Kim WS, Hodges JR, Piguet O, Halliday GM, Kiernan MC (2019). Eating peptides: biomarkers of neurodegeneration in amyotrophic lateral sclerosis and frontotemporal dementia. Ann Clin Transl Neurol.

[75] Xu Z, Lee A, Nouwens A, Henderson RD, McCombe PA (2018). Mass spectrometry analysis of plasma from amyotrophic lateral sclerosis and control subjects. Amyotroph Lateral Scler Front Degener.

[76] Steinacker P, Huss A, Mayer B, Grehl T, Grosskreutz J, Borck G, Kuhle J, Lulé D, Meyer T, Oeckl P, Petri S, Weishaupt J, Ludolph AC, Otto M (2017). Diagnostic and prognostic significance of neurofilament light chain NF-L, but not progranulin and S100B, in the course of amyotrophic lateral sclerosis: Data from the German MND-net. Amyotroph Lateral Scler Front Degener.

[77] Tremolizzo L, Pellegrini A, Conti E, Arosio A, Gerardi F, Lunetta C, Magni P, Appollonio I, Ferrarese C (2016). BDNF serum levels with respect to multidimensional assessment in amyotrophic lateral sclerosis. Neurodegener Dis.

[78] Dharmadasa T, Huynh W, Tsugawa J, Shimatani Y, Ma Y, Kiernan MC (2018). Implications of structural and functional brain changes in amyotrophic lateral sclerosis. Expert Rev Neurother.

[79] Strong MJ, Grace GM, Freedman M, Lomen-Hoerth C, Woolley S, Goldstein LH, Murphy J, Shoesmith C, Rosenfeld J, Leigh PN, Bruijn L, Ince P, Figlewicz D (2009). Consensus criteria for the diagnosis of frontotemporal cognitive and behavioural syndromes in amyotrophic lateral sclerosis. Amyotroph Lateral Scler.

[80] Montuschi A, Iazzolino B, Calvo A, Moglia C, Lopiano L, Restagno G, Brunetti M, Ossola I, Lo Presti A, Cammarosano S, Canosa A, Chio A (2015). Cognitive correlates in amyotrophic lateral sclerosis: a population-based study in Italy. J Neurol Neurosurg Psychiatry.

[81] Filippi M, Agosta F, Abrahams S, Fazekas F, Grosskreutz J, Kalra S, Kassubek J, Silani V, Turner MR, Masdeu JC, European Federation of Neurological Societies (2010). EFNS guidelines on the use of neuroimaging in the management of motor neuron diseases. Eur J Neurol.

[82] Schuster C, Kasper E, Dyrba M, Machts J, Bittner D, Kaufmann J, Mitchell AJ, Benecke R, Teipel S, Vielhaber S, Prudlo J (2014). Cortical thinning and its relation to cognition in amyotrophic lateral sclerosis. Neurobiol Aging.

[83] Masuda M, Senda J, Watanabe H, Epifanio B, Tanaka Y, Imai K, Riku Y, Li Y, Nakamura R, Ito M, Ishigaki S, Atsuta N, Koike H, Katsuno M, Hattori N, Naganawa S, Sobue G (2016). Involvement of the caudate nucleus head and its networks in sporadic amyotrophic lateral sclerosis-frontotemporal dementia continuum. Amyotroph Lateral Scler Front Degener.

[84] Agosta F, Ferraro PM, Riva N, Spinelli EG, Chiò A, Canu E, Valsasina P, Lunetta C, Iannaccone S, Copetti M, Prudente E, Comi G, Falini A, Filippi M (2016). Structural brain correlates of cognitive and behavioral impairment in MND. Hum Brain Mapp.

[85] Consonni M, Dalla Bella E, Contarino VE, Bersano E, Lauria G (2020). Cortical thinning trajectories across disease stages and cognitive impairment in amyotrophic lateral sclerosis. Cortex..

[86] Illán-Gala I, Montal V, Pegueroles J (2020). Cortical microstructure in the amyotrophic lateral sclerosis-frontotemporal dementia continuum. Neurology.

[87] Christidi F, Karavasilis E, Riederer F, Zalonis I, Ferentinos P, Velonakis G, Xirou S, Rentzos M, Argiropoulos G, Zouvelou V, Zambelis T, Athanasakos A, Toulas P, Vadikolias K, Efstathopoulos E, Kollias S, Karandreas N, Kelekis N, Evdokimidis I (2018). Gray matter and white matter changes in non-demented amyotrophic lateral sclerosis patients with or without cognitive impairment: a combined voxel-based morphometry and tract-based spatial statistics whole-brain analysis. Brain Imaging Behav.

[88] Branco LMT, de Rezende TJR, Roversi C d O (2018). Brain signature of mild stages of cognitive and behavioral impairment in amyotrophic lateral sclerosis. Psychiatry Res Neuroimaging.

[89] Machts J, Loewe K, Kaufmann J, Jakubiczka S, Abdulla S, Petri S, Dengler R, Heinze HJ, Vielhaber S, Schoenfeld MA, Bede P (2015). Basal ganglia pathology in ALS is associated with neuropsychological deficits. Neurology..

[90] Dimond D, Ishaque A, Chenji S, Mah D, Chen Z, Seres P, Beaulieu C, Kalra S (2017). White matter structural network abnormalities underlie executive dysfunction in amyotrophic lateral sclerosis. Hum Brain Mapp.

[91] Menke RAL, Proudfoot M, Talbot K, Turner MR (2018). The two-year progression of structural and functional cerebral MRI in amyotrophic lateral sclerosis. NeuroImage Clin.

[92] Trojsi F, Di Nardo F, Siciliano M et al (2020) Frontotemporal degeneration in amyotrophic lateral sclerosis (ALS): a longitudinal MRI one-year study. CNS Spectr 1–10. 10.1017/S109285292000005X 10.1017/S109285292000005X 32089134

[93] Trojsi F, Esposito F, de Stefano M, Buonanno D, Conforti FL, Corbo D, Piccirillo G, Cirillo M, Monsurrò MR, Montella P, Tedeschi G (2015). Functional overlap and divergence between ALS and bvFTD. Neurobiol Aging.

[94] Basaia S, Agosta F, Cividini C, Trojsi F, Riva N, Spinelli EG, Moglia C, Femiano C, Castelnovo V, Canu E, Falzone Y, Monsurrò MR, Falini A, Chiò A, Tedeschi G, Filippi M (2020). Structural and functional brain connectome in motor neuron diseases: a multicenter MRI study. Neurology..

[95] Hu T, Hou Y, Wei Q, Yang J, Luo C, Chen Y, Gong Q, Shang H (2020). Patterns of brain regional functional coherence in cognitive impaired ALS. Int J Neurosci.

[96] Nobili F, Arbizu J, Bouwman F, Drzezga A, Agosta F, Nestor P, Walker Z, Boccardi M, Festari C, Altomare D, Gandolfo F, Orini S, the EANM‐EAN Task Force for the Prescription of FDG‐PET for Dementing Neurodegenerative Disorders (2018). European Association of Nuclear Medicine and European Academy of Neurology recommendations for the use of brain 18 F-fluorodeoxyglucose positron emission tomography in neurodegenerative cognitive impairment and dementia: Delphi consensus. Eur J Neurol.

[97] Canosa A, Pagani M, Cistaro A, Montuschi A, Iazzolino B, Fania P, Cammarosano S, Ilardi A, Moglia C, Calvo A, Chiò A (2016). 18 F-FDG-PET correlates of cognitive impairment in ALS. Neurology..

[98] Buhour MS, Doidy F, Mondou A et al (2017) Voxel-based mapping of grey matter volume and glucose metabolism profiles in amyotrophic lateral sclerosis. EJNMMI Res 7(1). 10.1186/s13550-017-0267-2 10.1186/s13550-017-0267-2 PMC5339262 28266002

[99] Wicks P, Turner MR, Abrahams S, Hammers A, Brooks DJ, Leigh PN, Goldstein LH (2008). Neuronal loss associated with cognitive performance in amyotrophic lateral sclerosis: an (11C)-flumazenil PET study. Amyotroph Lateral Scler.

[100] Agosta F, Ferraro PM, Riva N, Spinelli EG, Domi T, Carrera P, Copetti M, Falzone Y, Ferrari M, Lunetta C, Comi G, Falini A, Quattrini A, Filippi M (2017). Structural and functional brain signatures of C9orf72 in motor neuron disease. Neurobiol Aging.

[101] Bede P, Bokde ALW, Byrne S, Elamin M, McLaughlin RL, Kenna K, Fagan AJ, Pender N, Bradley DG, Hardiman O (2013). Multiparametric MRI study of ALS stratified for the C9orf72 genotype. Neurology..

[102] Floeter MK, Danielian LE, Braun LE, Wu T (2018). Longitudinal diffusion imaging across the C9orf72 clinical spectrum. J Neurol Neurosurg Psychiatry.

[103] Walhout R, Schmidt R, Westeneng HJ, Verstraete E, Seelen M, van Rheenen W, de Reus MA, van Es MA, Hendrikse J, Veldink JH, van den Heuvel MP, van den Berg LH (2015). Brain morphologic changes in asymptomatic C9orf72 repeat expansion carriers. Neurology..

[104] Diehl-Schmid J, Licata A, Goldhardt O (2019). FDG-PET underscores the key role of the thalamus in frontotemporal lobar degeneration caused by C9Orf72 mutations. Transl Psychiatry.

[105] De Vocht J, Blommaert J, Devrome M (2020). Use of multimodal imaging and clinical biomarkers in presymptomatic carriers of C9orf72 repeat expansion. JAMA Neurol.

[106] Brettschneider J, Del Tredici K, Toledo JB (2013). Stages of pTDP-43 pathology in amyotrophic lateral sclerosis. Ann Neurol.

[107] Sporns O (2019) A cross-disorder connectome landscape of brain dysconnectivity. Nat Rev Neurosci 10.1038/s41583-019-0177-6 PMC8864539 31127193

[108] Benatar M, Turner MR, Wuu J (2019). Defining pre-symptomatic amyotrophic lateral sclerosis. Amyotroph Lateral Scler Front Degener.

[109] McMackin R, Bede P, Pender N, Hardiman O, Nasseroleslami B (2019). Neurophysiological markers of network dysfunction in neurodegenerative diseases. NeuroImage Clin.

[110] McMackin R, Dukic S, Broderick M, Iyer PM, Pinto-Grau M, Mohr K, Chipika R, Coffey A, Buxo T, Schuster C, Gavin B, Heverin M, Bede P, Pender N, Lalor EC, Muthuraman M, Hardiman O, Nasseroleslami B (2019). Dysfunction of attention switching networks in amyotrophic lateral sclerosis. NeuroImage Clin.

[111] Iyer PM, Mohr K, Broderick M, Gavin B, Burke T, Bede P, Pinto-Grau M, Pender NP, McLaughlin R, Vajda A, Heverin M, Lalor EC, Hardiman O, Nasseroleslami B (2017). Mismatch negativity as an indicator of cognitive sub-domain dysfunction in amyotrophic lateral sclerosis. Front Neurol.

[112] McMackin R, Dukic S, Costello E et al (2020) Localization of brain networks engaged by the sustained attention to response task provides quantitative markers of executive impairment in amyotrophic lateral sclerosis. Cereb Cortex 10.1093/cercor/bhaa076 PMC7391267 32318719

[113] Dukic S, McMackin R, Buxo T, Fasano A, Chipika R, Pinto-Grau M, Costello E, Schuster C, Hammond M, Heverin M, Coffey A, Broderick M, Iyer PM, Mohr K, Gavin B, Pender N, Bede P, Muthuraman M, Lalor EC, Hardiman O, Nasseroleslami B (2019). Patterned functional network disruption in amyotrophic lateral sclerosis. Hum Brain Mapp.

[114] Rossini PM, Burke D, Chen R, Cohen LG, Daskalakis Z, di Iorio R, di Lazzaro V, Ferreri F, Fitzgerald PB, George MS, Hallett M, Lefaucheur JP, Langguth B, Matsumoto H, Miniussi C, Nitsche MA, Pascual-Leone A, Paulus W, Rossi S, Rothwell JC, Siebner HR, Ugawa Y, Walsh V, Ziemann U (2015). Non-invasive electrical and magnetic stimulation of the brain, spinal cord, roots and peripheral nerves: basic principles and procedures for routine clinical and research application. An updated report from an IFCN Committee. Clin Neurophysiol.

[115] Agarwal S, Highton-Williamson E, Caga J, Matamala JM, Dharmadasa T, Howells J, Zoing MC, Shibuya K, Geevasinga N, Vucic S, Hodges JR, Ahmed RM, Kiernan MC (2018). Primary lateral sclerosis and the amyotrophic lateral sclerosis–frontotemporal dementia spectrum. J Neurol.

[116] Geevasinga N, Menon P, Özdinler PH, Kiernan MC, Vucic S (2016). Pathophysiological and diagnostic implications of cortical dysfunction in ALS. Nat Rev Neurol.

[117] Benussi A, Dell’Era V, Cantoni V, Cotelli MS, Cosseddu M, Spallazzi M, Micheli A, Turrone R, Alberici A, Borroni B (2020). TMS for staging and predicting functional decline in frontotemporal dementia. Brain Stimul.

